# Intragastric Localization as a Determinant of Peg Complications: A Comparative Analysis of Proximal and Distal Placements

**DOI:** 10.3390/medicina62010196

**Published:** 2026-01-17

**Authors:** Suat Evirgen, Şirin Çetin, Şencan Acar, Abdurrahman Şahin, Yavuz Pirhan, Hakan Sivgin, Meryem Çetin

**Affiliations:** 1 Department of General Surgery, Medical Faculty, Amasya University, Amasya 05100, Turkey; suat.evirgen@amasya.edu.tr; 2 Department of Biostatistics, Faculty of Medicine, Amasya University, Amasya 05100, Turkey; 3 Istanbul Florence Nightingale Hospital, Istanbul 34381, Turkey; 4 Gastroenterology Department, Faculty of Medicine, Tokat Gaziosmanpasa University, Tokat 60030, Turkey; 5 Department of General Surgery, Private Kolmed Hospital, Amasya 05100, Turkey; 6 Department of Internal Medicine, Gaziosmanpasa University Faculty of Medicine, Tokat 60100, Turkey; 7 Department of Microbiology, Faculty of Medicine, Amasya University, Amasya 05100, Turkey

**Keywords:** percutaneous endoscopic gastrostomy (PEG), intragastric localization, proximal placement, distal placement, complications

## Abstract

*Background and Objectives:* While percutaneous endoscopic gastrostomy (PEG) is a standard procedure for long-term enteral nutrition, the impact of precise intragastric tube localization on complications remains underexplored. This study aimed to determine whether proximal versus distal placement is a significant determinant of PEG-related complications and to identify associated risk factors. *Materials and Methods:* This retrospective study evaluated the medical records of 268 adult patients who underwent PEG for the first time at a single center between June 2022 and January 2025. Patients were divided into two groups based on the intragastric position of the PEG tube: Group A (proximal placement) and Group B (distal placement), defined anatomically in relation to the incisura angularis. The complication rate was 30.6% in patients with distally placed PEG tubes, compared to 14.1% in those with proximal placement. Demographic characteristics, PEG indications, body mass index (BMI), comorbidities, and anticoagulant use were recorded. Complications were classified as early (≤7 days) or late (8 days–6 months), and by severity as minor or major. *Results:* The complication rate was 30.6% in patients with distally placed PEG tubes, compared to 14.1% in those with proximal placement (*p* = 0.004), corresponding to an odds ratio of 2.7 (95% CI: 1.4–5.2). Both early and late complications, as well as minor and major events, were more frequently observed in the distal group. Patients with a low BMI and male patients demonstrated significantly higher co mplication rates (*p* = 0.0001 and *p* = 0.003). Five patients (1.8%) died due to PEG-related complications. *Conclusions:* PEG tubes positioned in the distal stomach carry a significantly higher risk of complications compared to proximal placement. These findings suggest that proximal intragastric positioning should be prioritized whenever feasible during PEG insertion to improve patient safety.

## 1. Introduction

Percutaneous endoscopic gastrostomy (PEG) is an essential and widely utilized technique for providing long-term enteral nutrition in patients who are unable to maintain adequate oral intake [1]. Since its introduction in 1980, PEG has largely replaced surgical gastrostomy in appropriate clinical settings, offering advantages such as the avoidance of general anesthesia, reduced hospital stay, and lower procedure-related morbidity [2]. Common indications include neurological dysphagia—caused by stroke, dementia, or Parkinson’s disease—as well as obstructive head and neck malignancies and other disorders impairing safe oral intake [3]. With the global rise in aging populations and the increasing prevalence of chronic neurological and malignant diseases, the clinical demand for PEG continues to grow [4].

Although PEG is considered minimally invasive and generally safe, it is not without risk. Reported complication rates vary widely in the literature, ranging from 10–15% in some series to over 40–50% in others [5]. Minor complications—such as peristomal infection, tube blockage, leakage, or granulation tissue formation—occur in 18–38% of cases, whereas major events, including aspiration pneumonia, gastrointestinal bleeding, visceral injury, or peritonitis, are observed in approximately 2–4% of patients [6]. Furthermore, 30-day mortality after PEG ranges from below 5% in general populations to over 20% in high-risk or malnourished cohorts [7].

Numerous studies have examined patient-related factors associated with PEG outcomes. A recent multicenter retrospective study by Stenberg et al. (2022) identified advanced age, female sex, diabetes mellitus, heart failure, low body mass index (BMI), and elevated C-reactive protein as predictors of increased complication and mortality risk following PEG [8]. However, despite these insights, a key procedural variable—the intragastric localization of the PEG tube—remains largely under-investigated. Specifically, it is unclear whether positioning the tube proximally (in the upper body of the stomach) versus distally (in the antrum) influences complication rates.

Several physiological mechanisms may plausibly explain that tube location might affect clinical outcomes. For example, increased peristaltic movement and thinner gastric walls in the distal stomach could predispose to mechanical complications or poor healing. Yet, to our knowledge, no previous study has directly compared complication rates between proximal and distal PEG placements.

This study had two primary objectives: (1) to evaluate whether the intragastric localization of the PEG tube is associated with differences in complication rates; and (2) to identify additional patient- and procedure-related factors contributing to adverse outcomes. We hypothesized that distal placement would be associated with a significantly higher rate of complications, particularly late-onset events, due to increased mechanical stress and anatomical disadvantages.

## 2. Materials and Methods

### 2.1. Study Design and Setting

This study was conducted using a retrospective cohort design at the Endoscopy Unit of Amasya University Şerefeddin Sabuncuoğlu Training and Research Hospital. Between June 2022 and January 2025, adult patients who underwent PEG placement were screened, and those meeting the inclusion criteria were enrolled for analysis.

### 2.2. Ethical Approval and Informed Consent

The study protocol was reviewed and approved by the Ethics Committee of Amasya University (Approval No: 2025/76). Due to the retrospective nature of the study, the requirement for individual informed consent was waived by the ethics committee.

### 2.3. Patient Selection

Inclusion Criteria: Patients were eligible if they met all of the following: 1—Age ≥ 18 years; 2—underwent first-time PEG placement during the study period; 3—had a clear clinical indication for long-term enteral nutrition, such as neurological dysphagia (e.g., stroke, dementia, Parkinson’s disease), malignancy-related obstructive dysphagia, or other disorders impairing safe oral intake; 4—had sufficient clinical data for assessment of intragastric PEG localization, complications, and follow-up.

Exclusion Criteria: Patients were excluded if they: 1—had a history of previous PEG placement or underwent PEG revision; 2—underwent major abdominal surgery simultaneously; 3—had incomplete or missing data; 4—were lost to follow-up before postoperative day 8 (except for mortality); 5—had contraindications to PEG, including active peritonitis, abdominal wall infection at the stoma site, or uncorrected coagulopathy; 6—were younger than 18 years of age.

### 2.4. PEG Procedure

PEG placement was performed using a 20 Fr. gastrostomy tube and the pull-through technique. All procedures were carried out under endoscopic guidance by a certified general surgeon and gastroenterologist. Antibiotic prophylaxis with 1 g of intravenous cefazolin was administered within 60 min before the procedure.

**Figure 1:**
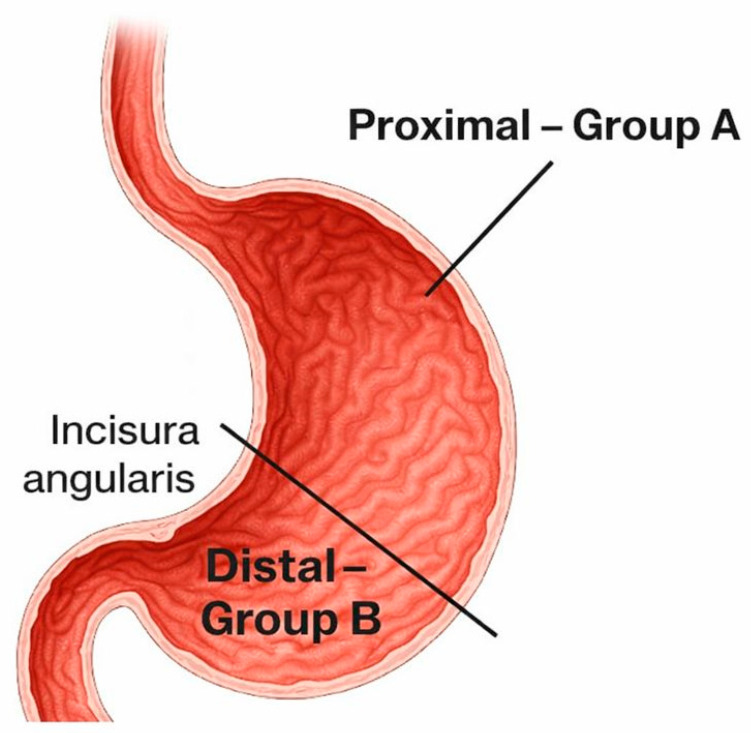
Anatomical boundary used to differentiate proximal and distal PEG placements.

During endoscopy, the site with optimal transillumination and visible digital indentation on the abdominal wall was selected. Based on the anatomical location of this optimal site, the PEG tube was placed either proximally or distally, depending on technical feasibility and patient-specific factors. Because the puncture site was selected according to the best transillumination/indentation window, group allocation was not randomized and may reflect anatomical and clinical factors that could confound the association between localization and complications. PEG localization was defined anatomically in relation to the incisura angularis: Group A (Proximal Placement): Tube positioned proximal to the incisura angularis. Group B (Distal Placement): Tube positioned distal to the incisura angularis (Figure 1). To enhance reproducibility, localization was classified using a predefined operational definition relative to the incisura angularis and documented in the endoscopy report using standardized terminology. When available, stored endoscopic images were reviewed during data extraction to support landmark identification.

The incisura angularis serves as the anatomical landmark separating proximal (Group A) from distal (Group B) placements. Proximal placements correspond to the gastric body and fundus, whereas distal placements are located in the antrum and pyloric region.

Following abdominal site preparation and local anesthesia with 2% lidocaine, a 25 G trocar needle was used for gastric puncture. A guidewire was inserted, retrieved through the mouth, and used to pull the PEG tube into position. External fixation was secured with a bumper, and tube placement was confirmed endoscopically. Patients began clear fluids after 12 h and were transitioned to enteral feeding if well tolerated.

### 2.5. Data Collection and Variables

Data were collected from hospital records and included demographic information, PEG indication, body mass index (BMI), comorbidities (e.g., diabetes, hypertension), anticoagulant use, and follow-up status.

Outcome measures were defined based on the frequency and type of PEG-related complications documented in hospital records. In accordance with the STROBE guidelines, complications were categorized by: Timing: Early (≤7 days) or Late (8 days–6 months), Severity: Minor or Major.

Minor complications were defined as events requiring minimal or no intervention and having limited clinical impact (e.g., peristomal leakage, mild local infection, tube dislodgement).

Major complications were defined as life-threatening or severe events requiring significant medical or surgical intervention (e.g., buried bumper syndrome, gastrointestinal bleeding, aspiration pneumonia, or necrotizing fasciitis).

### 2.6. Statistical Analysis

Statistical analyses were performed using SPSS version 25.0 (IBM Corp., Armonk, NY, USA). Continuous variables were expressed as mean ± standard deviation (SD) and compared using an independent samples *t*-test. Categorical variables were presented as counts and percentages, and assessed using the chi-square test where appropriate. Odds ratios (OR) and 95% confidence intervals (CI) were calculated to determine the strength of association between tube localization and complication rates. A *p*-value of <0.05 was considered statistically significant.

## 3. Results

### 3.1. Demographic Characteristics

The mean age of the 268 patients was 79.8 ± 3.7 years (mean ± SD). Of the study population, 152 (56.7%) were female and 116 (43.3%) were male. The mean age was 78.3 years in females and 82.1 years in males.

As anatomically defined in Figure 1, patients were categorized into proximal (Group A) and distal (Group B) PEG placement groups based on the position relative to the incisura angularis. There was no statistically significant difference in age between Group A (proximal placement) and Group B (distal placement) (independent samples *t*-test, *p* = 0.13). However, a significant difference in sex distribution was observed, with more females in Group A than in Group B (chi-square test, *p* = 0.036) (Table 1).

### 3.2. Complication Rates by Age and Sex

There was no significant association between age and complication development (79.8 ± 3.7 years in the non-complication group vs. 79.6 ± 3.8 years in the complication group; independent samples *t*-test, *p* = 0.471).

Male patients had significantly higher complication rates compared to female patients (chi-square test, *p* = 0.003). In Group A, this difference remained significant (chi-square test, *p* = 0.041) (Table 2).

### 3.3. Nutritional Status and Complication Risk

According to BMI classification, 51.1% of patients were underweight, 41.1% had normal weight, 4.9% were overweight, and 2.9% were obese (Table 3).

**Figure 2:**
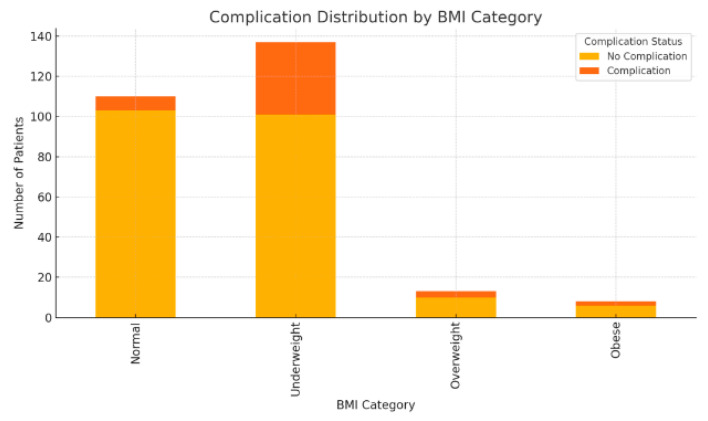
Complication Distribution by BMI Category.

There was a statistically significant association between BMI and complication rates (chi-square test, *p* < 0.001), mainly due to the elevated complication rate among underweight patients. Post hoc comparisons confirmed that underweight patients experienced significantly more complications than each of the other BMI categories. No significant difference was observed among normal, overweight, and obese groups (Table 4, Figure 2).

Among underweight patients, late complications were significantly more frequent than early ones (chi-square test, *p* < 0.0001; Table 5), and minor complications were more common than major ones (chi-square test, *p* < 0.001; Table 6).

Comparison of PEG-related complication occurrence across BMI categories. Complications include any early or late PEG-related adverse event during follow-up. Values are presented as the number of patients (n) in each BMI category and outcome group.

Distribution of early (≤7 days) and late (8 days–6 months) PEG-related complications by BMI category. Complications are classified according to timing after PEG placement. BMI categories are defined according to WHO criteria. Values are presented as the number of patients (n) in each BMI category and timing group.

Complications were more frequently observed in underweight patients, whereas normal-weight, overweight, and obese individuals showed markedly lower complication rates.

Comparison of the frequency of PEG-related minor and major complications across BMI categories. Minor complications include wound problems or peristomal leakage, whereas major complications include serious infection, bleeding, or organ injury. Values are presented as the number of patients (n) in each BMI category and severity group.

### 3.4. Indications and Comorbidities

PEG indications were neurological disorders (59.7%), malignancies (16.8%), and other causes (23.5%) (Table 7). There was no statistically significant association between indication type and complication development (chi-square test, *p* = 0.993).

Comorbid conditions included hypertension (64.2%), anticoagulant use (55.2%), smoking (42.9%), diabetes mellitus (25.4%), and COPD (21.3%). None of these variables were significantly associated with complication development (chi-square or Fisher’s exact test, *p* > 0.05).

Complication status according to primary indication for PEG placement (neurological causes, malignancy, or other causes). No statistically significant association was observed between PEG indication and complication occurrence (*p* = 0.993). Values are presented as the number of patients (n) in each indication and complication group.

### 3.5. Complication Rates by Intragastric Localization

Complications occurred in 48 patients (17.9%). Group B (distal placement) had a significantly higher complication rate (30.6%) than Group A (proximal placement) (14.1%) (chi-square test, *p* = 0.004). The odds of complication were 2.7 times higher in Group B (OR: 2.7; 95% CI: 1.4–5.2) (Table 8).

The table shows the distribution of overall complication status according to intragastric PEG localization (Group A vs. Group B). A significantly higher complication rate was observed in Group B (distal localization) compared with Group A (proximal localization) (*p* = 0.004). Values for Group A and Group B are presented as n (%) within each localization group; total values are given as follows.

n. Group A = proximal intragastric PEG placement; Group B = distal intragastric PEG placement; PEG = percutaneous endoscopic gastrostomy.

### 3.6. Early and Late Complication Rates

**Figure 3:**
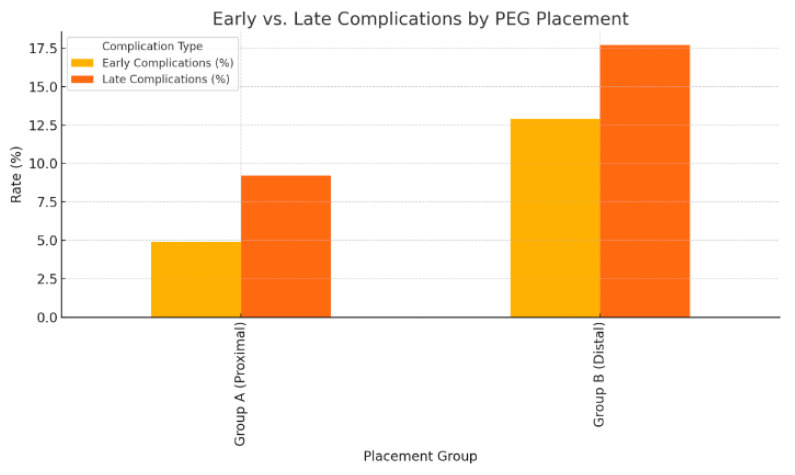
Early and late complication rates according to PEG placement site.

Early complications (≤7 days) occurred in 18 patients (6.7%), and late complications (8 days–6 months) occurred in 30 patients (11.2%). Both early (12.9% vs. 4.9%, *p* = 0.015) and late complications (17.7% vs. 9.2%, *p* = 0.008) were significantly more common in Group B than in Group A (chi-square test; Table 9, Figure 3).

The table shows a breakdown of early, late, minor, and major PEG-related complications between proximal (Group A) and distal (Group B) intragastric placements. *p*-values were calculated using chi-square or Fisher’s exact tests, as appropriate. Group B showed higher rates across all complication categories. Values are presented as n (%) within each PEG localization group; total values are given as n (% of the entire cohort).

Early and late complications were significantly more common in distal (Group B) PEG placements compared with proximal (Group A) placements, indicating a higher overall risk profile for distal localization.

### 3.7. Complications by Severity

Minor complications occurred in 33 patients (12.3%) and were significantly more frequent in Group B (17.7% vs. 10.7%, *p* = 0.007). Major complications were seen in 15 patients (5.6%), also more prevalent in Group B (12.9% vs. 3.4%, *p* = 0.001) (Table 10).

The table shows the distribution of minor PEG-related complications according to intragastric PEG localization (proximal vs. distal). Values are presented as n (%) within each PEG localization group; total values are given as n (% of the entire cohort).

### 3.8. Major Complications

The most common major complications included buried bumper syndrome (1.5%), aspiration pneumonia (1.1%), and necrotizing fasciitis (0.7%). Additional major events, such as intra-abdominal abscess or gastrointestinal bleeding, occurred in 2.0% of patients (Table 11).

Most cases of buried bumper syndrome (75%) and all cases of necrotizing fasciitis (100%) occurred in the distal group (Group B). A total of five PEG-related deaths (1.8%) were recorded during the study (Group A: n = 1; Group B: n = 4). Given the small number of events, this between-group distribution is reported descriptively and should be interpreted with caution.

The table shows the distribution of major PEG-related complications according to intragastric PEG localization (proximal vs. distal). Values are presented as n (%) within each PEG localization group; total values are given as n (% of the entire cohort). Major complications include buried bumper syndrome, necrotizing fasciitis, aspiration pneumonia, and other serious events.

## 4. Discussion

### 4.1. Comparison with Previous Literature

This study explored the impact of intragastric PEG tube localization—proximal versus distal—on complication rates, an aspect that remains under-investigated in the current literature. Our results demonstrated that PEG tubes placed in the distal stomach were associated with significantly higher complication rates compared to proximal placements. These findings are consistent with our initial hypothesis and suggest that the anatomical site of PEG insertion may be associated with clinical outcomes.

Previous literature has largely focused on patient-related risk factors, with little attention to procedural anatomy [9]. By identifying the antrum as a higher-risk site, our study offers novel insights that may redefine risk stratification in PEG procedures. Both early and late complications, as well as minor and major adverse events, were significantly more frequent in the distal placement group, reinforcing the importance of tube localization.

Similarly, a recent systematic review and meta-analysis by Farrugia et al. (2024) [10] found that the most common complications following gastrostomy were peristomal infections and unintended tube replacements, which remained consistent across various patient populations and procedural settings. These findings are in line with our results, where peristomal cellulitis (6.3%) and tube dislodgement (1.5%) emerged as the most frequent minor complications, particularly in patients with distal tube placement. Farrugia’s data support the idea that certain complications are not solely patient-dependent but may also be influenced by technical factors such as tube positioning [10].

### 4.2. Patient Characteristics and Risk Factors

Conventional predictors such as advanced age, PEG indication, and common comorbidities (e.g., diabetes, hypertension, or malignancy) were not significantly associated with complication development. This finding challenges some established assumptions in the literature and highlights the potential limitations of traditional risk models that overlook anatomical and procedural variables.

Regarding PEG indications, we found no significant difference in complication rates among patients with neurological, oncological, or other conditions. While prior studies, including those focusing on head and neck cancers, suggested higher complication risks in malignancy cases, our results align with those of Grant et al. [11], emphasizing that nutritional and clinical status may be more critical than diagnosis alone.

Our findings also revealed that male sex was significantly associated with higher complication rates. Although the exact mechanism remains unclear, factors such as differences in body composition, abdominal wall thickness, and tissue elasticity may contribute. Similar associations have been reported by Stenberg et al. [8], reinforcing the need for further gender-based anatomical investigation.

### 4.3. Nutritional Status and Clinical Significance

Nutritional status emerged as a key determinant of outcomes. Patients with low BMI had a significantly increased risk of both minor and late complications. This association may reflect impaired wound healing, reduced immune competence, and compromised tissue resilience among undernourished individuals. These findings are in line with prior work by Sousa et al. [12], highlighting the importance of pre-procedural nutritional assessment and optimization.

A recent meta-analysis by Al-Salihi et al. (2025) [13] emphasized the critical role of undernutrition in predicting PEG-related adverse events, particularly in stroke patients. The study showed that malnourished individuals had significantly higher rates of minor and late-onset complications. These conclusions resonate closely with our results: patients with low BMI (<18.5) had the highest complication rates in our cohort, especially minor (24/33) and late (20/30) events. This reinforces the notion that nutritional status is a central prognostic variable in PEG outcomes, regardless of the patient’s primary diagnosis [13].

### 4.4. Mechanistic Insights: Why Distal Placement Carries Higher Risk

The most striking and original finding in this study is the nearly 2.7-fold increase in complication risk with distal PEG placement. This trend was consistently observed across all types of complications.

This observation aligns with the latest guidelines from the American Society for Gastrointestinal Endoscopy (ASGE), which recommend caution when considering distal PEG tube placement. According to ASGE, distal stomach positioning—especially near or beyond the pylorus—may increase the risk of aspiration, delayed gastric emptying, and tube dislodgement. Therefore, optimal positioning should be guided by individual anatomical and procedural factors, with a preference for proximal sites when feasible to reduce complication risks [14].

Several plausible mechanisms may underlie this observation. The antrum is subject to more intense peristaltic motion and pyloric activity, which may increase mechanical stress on the internal bumper and predispose to buried bumper syndrome (BBS). Additionally, the antral wall is thinner and more mobile, offering less stability and a higher likelihood of ischemic injury. From a technical standpoint, distal sites also present greater challenges in achieving optimal transillumination and digital indentation, potentially increasing procedural risk.

### 4.5. Clinical Implications

These findings carry direct relevance for clinical practice. First, nutritional status must be evaluated thoroughly before PEG placement, especially in underweight patients who are inherently more vulnerable to complications. Second, and most importantly, proximal placement should be prioritized whenever anatomically feasible. Selecting a site in the anterior gastric body may significantly reduce complication rates and improve overall safety.

In our cohort, PEG-related mortality was 1.8% (5/268 patients), a rate that falls within the range reported in recent series. Arslan et al. [15] described a PEG-related mortality of 1.7% in patients with head and neck cancer, which is strikingly similar to our overall findings. Four of the five deaths occurred in patients with distally positioned tubes; however, mortality events were rare, and the study was not powered for mortality comparisons between groups. Therefore, this observation should be considered descriptive and hypothesis-generating rather than confirmatory.

We recommend that intragastric localization be considered a deliberate and modifiable factor during PEG planning—not just a procedural detail. This may ultimately help refine patient selection, improve outcomes, and reduce PEG-related morbidity and mortality.

### 4.6. Limitations and Future Directions

This study has certain limitations. First, its retrospective, non-randomized design introduces inherent biases and limits causal inference. Second, the single-center nature of the study may affect the generalizability of results to broader patient populations. Because localization relied on endoscopic landmark recognition, inter-operator variation and formal reproducibility (e.g., inter-rater agreement) were not quantified in this retrospective dataset and should be evaluated in future prospective studies.

Additionally, multivariate logistic regression analysis could not be performed to control for potential confounding variables such as chronic kidney disease (CKD), congestive heart failure (CHF), use of anticoagulants, INR levels, and platelet count. The primary reason for this was the retrospective nature of the study and the lack of complete and consistent data for these variables across all patients. Conducting multivariate analysis with missing or heterogeneous data may lead to biased or misleading results. Therefore, only univariate statistical evaluations were performed in this study, and this methodological limitation should be considered when interpreting the findings.

Importantly, localization was determined by technical feasibility rather than random allocation. Patients requiring distal placement may have had more challenging anatomy or clinical vulnerability (e.g., limited proximal transillumination due to prior abdominal surgery, deformity/kyphoscoliosis, altered gastric position, or increased frailty), which could independently increase complication risk. As these factors were not systematically captured and adjusted for, residual confounding and selection bias cannot be excluded; therefore, our findings should be interpreted as associative rather than causal.

Nonetheless, the relatively large sample size and standardized procedural protocol lend strength to our conclusions.

Future studies should aim to address these limitations through prospective, multi-center randomized trials. Furthermore, research incorporating advanced imaging and biomechanical analysis could better elucidate anatomical differences and procedural dynamics. Investigating gastric wall thickness, pyloric mobility, and internal tension via imaging may enhance understanding of how tube positioning impacts complication risk. Lastly, sex-specific anatomical differences should be further explored to develop personalized procedural strategies.

## 5. Conclusions

This study highlights a critical and previously overlooked factor in PEG procedures: the intragastric localization of the tube. Our results suggest that the anatomical site of PEG placement significantly influences the frequency and severity of post-procedural complications.

Distal placements, particularly in the antrum, were associated with approximately 2.7-fold higher odds of both minor and major complications compared to proximal placements. These findings suggest that, in addition to how PEG is performed, where the tube is positioned may be clinically relevant for patient safety.

Contrary to prior assumptions, traditional risk factors such as advanced age, PEG indication, or diabetes mellitus did not significantly affect complication rates in our cohort. In contrast, low body mass index (BMI) and male sex were significantly associated with an increased risk of complications in univariate analyses. These findings emphasize the need for individualized pre-procedural assessment, especially in underweight or male patients.

Procedure-related mortality was low (1.8%). Given the small number of deaths, no firm conclusions regarding mortality differences by localization can be drawn.

**Clinical Implications:** Proximal positioning within the gastric body should be prioritized whenever anatomically feasible. A systematic approach to tube localization, rather than relying solely on endoscopic transillumination or technical ease, may reduce complication risk. In addition, malnourished patients may benefit from pre-procedural nutritional support.

In summary, intragastric tube placement is a modifiable procedural variable that, in this cohort, was associated with differences in outcomes. These findings support considering anatomical positioning during procedural planning, while definitive protocol changes should be confirmed in prospective and/or adjusted studies.

## Figures and Tables

**Table 1:** Baseline Demographic Characteristics of the Study Population.

Characteristic	Group A (n = 206)	Group B (n = 62)	*p*-Value
Age (mean ± SD)	79.61 ± 3.59	80.42 ± 3.93	0.130
Gender (F/M)	124/82	28/34	0.036

Baseline age and sex distribution in patients with proximal (Group A) and distal (Group B) PEG tube placement. Group A = proximal intragastric PEG placement; Group B = distal intragastric PEG placement; SD = standard deviation.

**Table 2:** Association Between Sex and Complication Development.

Sex	No Complication	Complication Present	Total
Female	134 (60.9%)	18 (37.5%)	152 (56.7%)
Male	86 (39.1%)	30 (62.5%)	116 (43.3%)
Total	220	48	268

Distribution of PEG-related complications according to sex in the overall study cohort. Complications include any PEG-related adverse event occurring during follow-up. Values are given as n (%) within each outcome column.

**Table 3:** Body Mass Index (BMI) Categories of the Study Population.

Category	BMI (kg/m^2^)	Number of Patients	Percentage
Normal	18.5–24.9	110	41.1%
Underweight	<18.5	137	51.1%
Overweight	25.0–29.9	13	4.9%
Obese	≥30.0	8	2.9%

Body mass index (BMI) distribution of all patients included in the study. BMI categories were defined according to WHO criteria: underweight (<18.5 kg/m^2^), normal (18.5–24.9 kg/m^2^), overweight (25.0–29.9 kg/m^2^), and obese (≥30.0 kg/m^2^). Percentages indicate the proportion of the entire study cohort.

**Table 4:** Relationship Between Body Mass Index and Complication Development.

BMI Category	No Complication	Complication Present	Total
Normal	103	7	110
Underweight	101	36	137
Overweight	10	3	13
Obese	6	2	8
Total	220	48	268

A significant difference was found between BMI categories and complication rates (Chi-square test, *p* = 0.0007).

**Table 5:** Association Between Complication Timing and BMI Categories.

Complication Timing	Normal	Underweight	Overweight	Obese	Total
No Complication	103	101	10	6	220
Early	2	16	0	0	18
Late	5	20	3	2	30
Total	110	137	13	8	268

A significant association was observed between BMI categories and complication timing (Chi-square test, *p* = 0.0011).

**Table 6:** Severity of Complications According to BMI Categories.

Complication Severity	Normal	Underweight	Overweight	Obese	Total
No Complication	103	101	10	6	220
Minor	4	24	3	2	33
Major	3	12	0	0	15
Total	110	137	13	8	268

A statistically significant association was found between BMI categories and complication severity (Chi-square test, *p* = 0.0025).

**Table 7:** Relationship Between PEG Indications and Complication Development.

Complication Status	Neurological Causes	Malignancy	Other Causes	Total
No complication	131	37	52	220
Complication present	29	8	11	48
Total	160	45	63	268

No significant association was found between PEG indications and complication development (Chi-square test, *p* = 0.993).

**Table 8:** Overall Complication Rates by Intragastric PEG Localization.

Complication Status	Group A (n = 206)	Group B (n = 62)	Total (n = 268)
No complication	177(85.9%)	43 (69.4%)	220
Complication	29(14.1%)	19(30.6%)	48
Total	206	62	268

A significant difference in complication rates was found between Group A and Group B (Chi-square test, *p* = 0.004).

**Table 9:** Detailed Comparison of Complication Types Between Groups A and B.

Outcome	Group A (n = 206)	Group B (n = 62)	Total (n = 268)	*p*-Value
Any complication	29 (14.1%)	19 (30.6%)	48 (17.9%)	0.004
Early (≤7 days)	10 (4.9%)	8 (12.9%)	18 (6.7%)	0.015
Late (8 days–6 months)	19 (9.2%)	11 (17.7%)	30 (11.2%)	0.008
Minor	22 (10.7%)	11 (17.7%)	33 (12.3%)	0.007
Major	7 (3.4%)	8 (12.9%)	15 (5.6%)	0.001

All *p*-values were calculated using Chi-square or Fisher’s exact tests and were statistically significant (*p* < 0.05).

**Table 10:** Distribution of Minor Complications by PEG Localization.

Complication Type	Group A (n = 206)	Group B (n = 62)	Total (n = 268)
Peristomal cellulitis	11 (5.3%)	6 (9.7%)	17 (6.3%)
Peristomal leakage	7 (3.4%)	3 (4.8%)	10 (3.7%)
PEG tube dislodgement	3 (1.5%)	1 (1.6%)	4 (1.5%)
PEG tube occlusion	0 (0.0%)	1 (1.6%)	1 (0.4%)
Local bleeding/hematoma	1 (0.4%)	0 (0.0%)	1 (0.4%)

No statistically significant difference was found in the distribution of minor complication types between groups (Chi-square test, *p* = 0.6084).

**Table 11:** Distribution of Major Complications by PEG Localization.

Complication Type	Group A (n = 206)	Group B (n = 62)	Total (n = 268)	*p*-Value
Buried bumper syndrome	1 (0.5%)	3 (4.8%)	4 (1.5%)	0.045
Necrotizing fasciitis	0 (0.0%)	2 (3.2%)	2 (0.7%)	0.018
Aspiration pneumonia	3 (1.5%)	0 (0.0%)	3 (1.1%)	0.554
Other major complications	2 (1.0%)	3 (4.8%)	5 (2.0%)	0.082

Buried bumper syndrome and necrotizing fasciitis occurred significantly more often in Group B (*p* = 0.045 and *p* = 0.018, respectively). Other major complications did not show statistically significant differences between groups (*p* > 0.05).

## Data Availability

The data presented in this study are available from the corresponding author upon reasonable request. Due to ethical and privacy concerns, the dataset is not publicly accessible.
